# International approaches to rural generalist medicine: a scoping review

**DOI:** 10.1186/s12960-018-0332-6

**Published:** 2018-11-21

**Authors:** Nicholas Schubert, Rebecca Evans, Kristine Battye, Tarun Sen Gupta, Sarah Larkins, Lachlan McIver

**Affiliations:** 1 James Cook University, Townsville, Australia; 2 College of Medicine and Dentistry, James Cook University, Townsville, Australia; 3 KBC, Orange, Australia; 4 Rocketship Pacific Ltd, Geneva, Switzerland

**Keywords:** Rural, Remote, Medicine, Generalism, Primary health care

## Abstract

**Background:**

Contemporary approaches to rural generalist medicine training and models of care are developing internationally as part of an integrated response to common challenges faced by rural and remote health services and policymakers (addressing health inequities, workforce shortages, service sustainability concerns). The aim of this study was to review the literature relevant to rural generalist medicine.

**Methods:**

A scoping review was undertaken to answer the broad question ‘What is documented on rural generalist medicine?’ Literature from January 1988 to April 2017 was searched and, after final eligibility filtering (according to established inclusion and exclusion criteria), 102 articles in English language were included for final analysis.

**Results:**

Included papers were analysed and categorised by geographic region, study design and subject themes. The majority of articles (80%) came from Australia/New Zealand and North America, reflecting the relative maturity of programmes supporting rural generalist medicine in those countries. The most common publication type was descriptive opinion pieces (37%), highlighting both a need and an opportunity to undertake and publish more systematic research in this area.

Important themes emerging from the review were:

- Definition
- Existing pathways and programmes
- Scope of practice and service models
- Enablers and barriers to recruitment and retention
- Reform recommendations

There were some variations to, or criticisms of, the definition of rural generalist medicine as applied to this review, although this was only true of a small number of included articles. Across remaining themes, there were many similarities and consistent approaches to rural generalist medicine between countries, with some variations reflecting environmental context and programme maturity. This review identified recent literature from countries with emerging interest in rural generalist medicine in response to problematic rural health service delivery.

**Conclusions:**

Supported, coordinated rural generalist medicine programmes are being established or developed in a number of countries as part of an integrated response to rural health and workforce concerns. Findings of this review highlight an opportunity to better share the development and evaluation of best practice models in rural generalist medicine.

## Background

A rural generalist, for the purpose of this review, is defined as a community physician, primary care physician, general practitioner (GP), or family practitioner/family physician, with ‘recognised skill sets and qualifications, credentialed to provide primary care, hospital, emergency and population health care as well as one or more areas of advanced specialised practice in a rural, remote and/or regional setting’ [1]. This definition is consistent with the Cairns Consensus Statement (May 2014), an international document defining rural generalist medicine (RGM) and its key pillars and supported by representatives of the First World Summit on Rural Generalist Medicine in 2013. The Cairns Consensus Statement describes RGM as ‘the provision of a broad scope of medical care by a doctor in the rural context that encompasses the following:

- Comprehensive primary care for individuals, families and communities;
- Hospital in-patient and/or related secondary medical care in the institutional, home or ambulatory setting;
- Emergency care;
- Extended and evolving service in one or more areas of focused cognitive and/or procedural practice as required to sustain needed health services locally among a network of colleagues;
- A population health approach that is relevant to the community;
- Working as part of a multi-professional and multi-disciplinary team of colleagues, both local and distant, to provide services within a ‘system of care’ that is aligned and responsive to community needs’ [2].

Contemporary RGM must be considered against a backdrop of challenges faced by policymakers, health services and medical educators in addressing ongoing health inequities [3], workforce shortages [4] and service sustainability concerns specific to rural and remote areas around the world [5, 6]. These challenges reflect the paradox of the ‘inverse care law’ and the inequity of access to health care in areas of most need; in this case rural and remote communities [7]. More recently, a number of countries have investigated RGM as part of an integrated solution to these issues, including supported pathways aimed at developing a rural medical workforce skilled in primary health care, public health and advanced specialist care [1, 8]. This emerging international focus on RGM is highlighted by three RGM World Summits since 2013; now a biennial event [2, 9].

RGM has been a feature of medicine in countries with large rural and/or remote areas for a considerable time [8, 10], despite variations in rural generalist titles, nature of training programmes and models of care. However, the commitment to coordinated RGM training is now occurring in a climate of generalist practitioner shortages [11], most prevalent in rural communities and areas of socio-economic disadvantage [12–15]. Rural workforce shortages have been identified by the World Health Organization (WHO) as a significant barrier to universal, equitable health coverage [16]. Some of the common drivers for these shortages include the increasing trend toward metropolitan-based medical specialisation [17]; feminisation and ageing of the medical workforce; changing work priorities of younger doctors; changing attitudes toward owning a general practice; and, negative perceptions of both rural and general practice [18, 19].

This scoping review aims to capture, analyse and summarise the international state of knowledge relevant to the development and support of RGM training, models of care and clinical practice.

## Methods

The question ‘what is documented on rural generalist medicine?’ ensured that a broad range of literature was captured in this review. Broad analysis of the scale and scope of available literature is consistent with scoping review methodology and the five stage framework developed by Arksey and O’Malley: establishing the research question, identifying relevant studies, selecting studies to be included, charting data and summarising results [20].

Inclusion and exclusion criteria (Table 1) focused the search results to ensure relevance of findings. Government and education policies aimed at addressing the geographic maldistribution of the medical workforce took a significant shift from the late 1980s and continued during the 1990s [19, 21, 22]. To capture this change, literature from January 1988 to April 2017 was sourced and reviewed.

**Table 1:** The inclusion/exclusion criteria applied to the screening of the papers for this review

Criterion	Inclusion	Exclusion
Time period	January 1988 to April 2017	Studies outside of these dates
Language	English	Non-English studies
Type of article	No limits were placed on the literature type	N/A
Study focus	Rural medical generalism: the definition and scope of rural medical generalists, the RMG training pathway, enablers and barriers to practise and recommendations for reform Rural generalism definition as general practitioner/family practitioner/family physician in rural areas with reference to provision of primary and emergency care and one or more specialised practice The definition of rural generalist medicine involves an inclusive use of the term ‘rural’, to reflect the context of the specific research setting	Articles on rural generalism that did not meet the specific definition of this paper Disciplines outside of medicine (allied health, nursing and health support roles) Rural specialist physicians including ‘generalist specialists’ Articles with a focus only on a specific advanced skill (e.g. obstetrics) unless it was examined in the context of rural generalism Disease specific research
Literature focus	Articles with specific reference to the development of rural medical generalism and/or rurally based general practitioners, family practitioners, primary health medical practitioners or family physicians with specialised (procedural or non-procedural) skills	Articles that discussed rural health, rural medicine and/or rural GPs but without reference to rural medical generalism as defined here
Population and sample	Rurally based medical generalists as rural GPs/primary medical care providers with specialised skills (including rural GP proceduralists)	Other GPs and/or medical practitioners outside of the definition of rural medical generalist

Medical subject headings (MeSH) and Boolean operators were used to narrow, widen and combine literature searches and ensure relevant literature was captured in the search Table 2). This search was supplemented by bibliographic searching and inclusion of grey literature.

**Table 2:** Search terms and databases

Search terms and databases	
Rural* AND Generalist* OR Generalism* (Informit) Rural* AND Generalist* OR Generalism* (PubMed) Rural* AND Generalism* OR Generalist* (CINAHL) Rural* AND Generalist* OR Generalism* (MEDLINE and EMBASE)	
Other search methods	
• Manual search of reference lists of identified articles • Google Scholar • Grey literature from existing resources known and identified from specific grey literature data searches (on Trove, La Trobe Library Search, Scopus and Web of Science).	

* used as a wildcard to broaden the search term

**Fig. 1:**
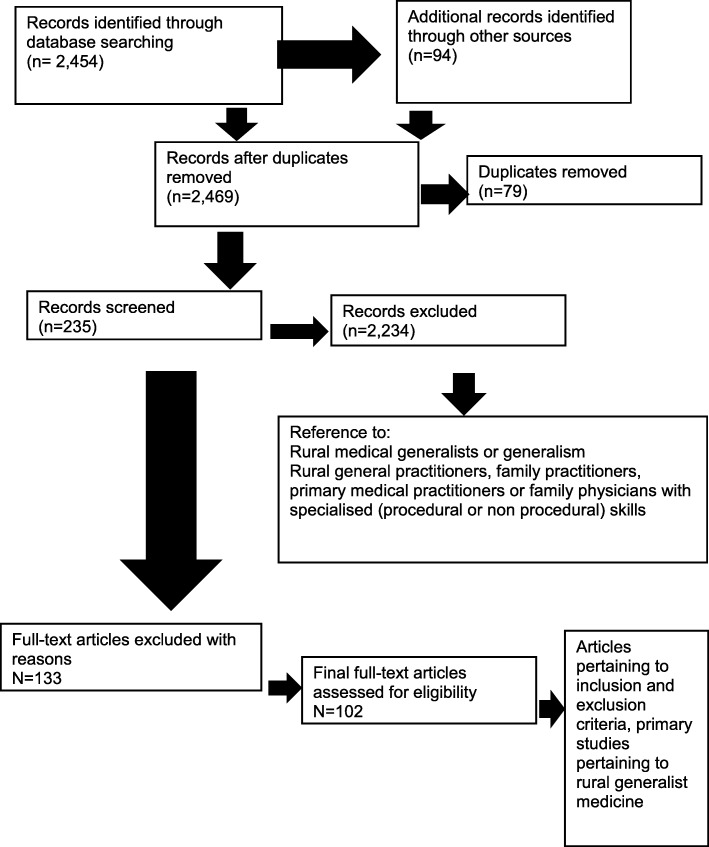
Overview of the review process

Using these parameters, 2454 articles were initially retrieved in the database searches. The variation in titles and terms within the RGM field may have had some influence on the search results. However, after selecting the relevant articles based on inclusion criteria (Table 1) and removing duplicates, 140 articles were retained for review (Fig. 1). A further 36 articles were identified using Google Scholar. Grey literature obtained through data searches and prior knowledge added another 39 articles (this included 11 websites). A further 17 articles were included as a result of bibliographic searching. Three more articles were included as a result of manual journal searches. Guided by the inclusion and exclusion criteria, a total of 235 studies were identified as relevant to the research topic.

During the final article selection, more were excluded on the grounds of relevance. In some cases, these articles did focus on ‘generalism’ but not rurality; others focused on the rural health workforce but without reference to generalism as defined in this review (Fig. 1). After final eligibility filtering, 102 articles were included in this review (Fig. 1 and Table 3). Another author reviewed all articles for consensus on inclusion.

**Table 3:** Sources of retrieved and included papers

Source	Retrieved	Included
Databases (MEDLINE, CINAHL, Informit Health, EMBASE and PubMed)	140	44
Google Scholar	36	20
Grey Literature	39	24
Snowballing	17	14
Journal searches	3	0
Total	235	102

## Results

Articles included for the final review were identified by geographic origins to enable a comparative analysis of rural generalist medicine data by region (Table 4).

**Table 4:** Geographic regions of included papers

Region	Included
Asia Pacific	4
Australia/New Zealand	46
North America	36
Africa	11
Europe	4
International (WHO)	1
Total	102

Articles were also identified by type of article or design (some were combined approaches, explaining the total greater than 102; Table 5). The majority were descriptive opinion articles.

**Table 5:** Articles by report type

Report type	Article numbers
Descriptive opinion piece	40
Quantitative data analysis	22
Qualitative study (e.g. interviews/focus groups)	12
Position paper	10
Literature review	8
Programme description	8
Government report	7
Systematic review	2
Total	109

Table 6 contains a summary of each article by region, including the main findings. Data extracted from the articles was coded into key themes, including:

- Definition of rural generalism
- Existing pathways and programmes
- Scopes of practice and service models
- Enablers and barriers to recruitment and retention
- Reform Recommendations

**Table 6:** Summary of included papers by region

Author (citation number)	Location	Year	Study design/methods/sample	Findings/critique
Asia Pacific				
Basnyat [101]	Nepal	2013	Descriptive opinion piece	• Outlines main emergency procedures of working in Kalikot District Hospital, including surgery and obstetrics procedures required of the primary care doctors and the resource limitations that they face.
Matsumoto et al. [98]	Japan	2005	Quantitative study—survey	• From this survey the only factor relevant to training identified as ‘retention enhancing’ has been family medicine training. • Authors advocate for an identified rural generalist practitioner training programme with a broad skill base with identified advanced skills (e.g. internal medicine, gastroenterology, general surgery). Obstetrics in rural Japan are however covered by specialists.
Mavalankar et al. [102]	India	2009	Qualitative study—interviews	• Shortage of anaesthetists in India—especially in rural areas—and the programme developed to train MOs in LSAS. Highlighted protectionist opposition to the programme. • 14 surveyed reported the practical training was too short. 5 felt that the training had not sufficiently prepared them. Training programme duration should be extended and that more time spent on practical areas.
Moore [100]	Nepal	2006	Descriptive opinion piece	• Overview of critical health needs in Nepal • Highlights the need for a generalist workforce specific to Nepal, especially in subsistence farmland areas.
Australia/New Zealand				
ACRRM [76]	Australia	2002	Literature review and quantitative study—survey	• Outlines a decline in the rural procedural workforce and the barriers to practise that have impacted on this decline.
ACRRM [86]	Australia	2002	Position paper	• Provides recommendations to address the barriers to procedural practice outlined in the research report.
ACRRM [25]	Australia	2014	Position paper	• Identifies key issues, enablers and barriers to establishing streamlined training and education for a career in Rural Generalist Medicine. • Proposes a national officer position within the Commonwealth Department be established to oversee the integration of national RG pathway. Also has 28 Recommendations for reform to build and support the RG pathway.
ACRRM [2]	Australia	2014	Position paper	• Statement with international endorsement of a definition and recommendations for rural generalist medicine. The definition provided involves an integrated model of both primary and secondary care and recommendations are provided under the domains of Recognition, Training Pathways and Research.
ACRRM, RDAA and ARRWAG [103]	Australia	2002	Position paper	• Provides recommendations to governments on key rural generalist reform areas.
AMA [104]	Australia	2012	Position paper	• Supports enhanced generalist pathways and greater recognition, including RGs.
AMA [105]	Australia	2014	Position paper	• Regional Training Networks needed to establish expanded generalist and specialist post graduate training positions in regional/rural areas. At present Rural Generalist and Specialist Training programmes are the only supported programmes addressing regional long-term placement in postgraduate medical training.
Carson [106]	Australia	2009	Descriptive opinion piece	• Consider cooperative models of remote (surgical) specialist delivery in the Northern Territory, delivered by appropriately trained generalists in cooperation with and supported by specialists from larger areas.
Department of Health and Human Services, Tasmania [51]	Australia	Web link 2016	Programme description	• Overview of Tasmanian RG pathway and describes the RMG as ‘a medical practitioner who is trained, mentored and supported on a ‘career superhighway’.
Department of Health, Victoria [28]	Australia	2014	Government report	• Provides a definition of a RG and characteristics of the Victorian RG programme.
Ellis and Philip [84]	Australia	2010	Descriptive opinion piece	• Describes the need for priority up-skilling generalist clinicians to meet the needs of clients facing mental health emergencies in rural and remote areas. • Proposes the use of the ‘Mental Health Emergencies’ training programme developed by the Australian Rural Nurses and Midwives based on the success of this programme.
Glazebrook and Harrison [75]	Australia	2006	Literature review	• Identifies the barriers to the maintenance of advanced procedural skills for rural generalists.
Government of South Australia: SA Health [107]	Australia	Web link 2016	Programme description	• Highlights AST training support for GPs in anaesthetics and obstetrics.
Hays et al. [108]	Australia	2005	Qualitative study—interviews	• The analysis highlights the differing views between health professionals and rural patients on the quality of care in rural hospitals. • Patients and families focussed on interpersonal skills whereas health professionals focussed on workforce and technical aspects of care. • All groups agreed on the need to be able to continue to provide the flexible care and familiar surrounds offered by proceduralists in small rural hospitals. • This study indicates the ongoing need for rural health professionals to be trained in providing procedural medical care in smaller rural hospitals, despite the developing trend to centralise procedural tasks in larger urban areas.
Health Education and Training Institute (HETI) [26]	Australia	Web link 2016	Programme description	• Provides definition and overview of the NSW RG pathway. • Supported advanced skills are anaesthetics, obstetrics and ‘advanced skill set’ (Obstetrics and Emergency Medicine).
HWA [109]	Australia	2012	Government report	• Overview of the imbalance in the distribution of the specialist vs generalist medical workforce. • Identifies concerns about the decline in generalists and impact of rural workforce. • Also identifies the geographic maldistribution across the total medical specialties including general practice.
HWA [1]	Australia	2013	Government report	• Background to the need to focus on supporting the RMG pathway. Reform recommendations across six domains.
HWA [77]	Australia	2014	Quantitative study—survey	• Shows that over half GP proceduralists in rural Victoria are not using their procedural skills and 32% plan to retire in the next 5 years. Identifies key barriers to practise.
Humphreys et al. [67]	Australia	2003	Quantitative study—survey	• Finds that the proportion of GPs providing complex services increases with increasing rurality or remoteness.
Jones [65]	Australia	2014	Descriptive opinion piece	• Posits generalism as the domain of all general practitioners, with the changing role of rural based generalists. • Outlines training programme for rural generalist of the RACGP.
Kitchener [50]	Australia	2013	Descriptive opinion piece	• Overview of the challenges and issues arising from the Queensland RGP and argues for regional training providers to address some of the training issues regarding private and public placements.
Larkins and Evans [73]	Australia	2014	Descriptive opinion piece	• Advocates policy support for Rural Generalists.
Lawrance [39]	Australia	2007	Literature review	• Questions the use of term ‘rural generalism’ in Australia based on the US model of community based training programmes. • The main application for the term in Australia, then, is to describe a state government (Qld) hospital role. The author claims this is a government definition for a government purpose.
Lee [38]	Australia	2015	Descriptive opinion piece	• Provides an historical account of the evolution of generalism and an overview of the different definitions. • Supports the development of a definition that embraces the diversity of approaches and settings in generalism and argues that a political approach is needed to revive the generalist profession.
Manahan et al. [23]	Australia	2011	Programme description (conference paper)	• Background discussion on QRGP and the outcomes of the research into the advanced skills. • Also raises the concept of the rural generalist as applicable in other jurisdictions, and perhaps in other disciplines.
Mason [88]	Australia	2013	Government (commissioned) report	• Proposes a new rural training pathway to support the training of both rural generalist specialists and rural generalist GPs. The proposed model focusses on a regionally coordinated programme from undergraduate through to fellowship.
McKenzie et al. [110]	Australia	2013	Quantitative study—survey	• Overall this study of outcomes from advanced rural skills training in Qld has shown that the majority of GPs and Rural Generalists are using their advanced procedural and non-procedural skills but also that there is room for improvement. • Unlikely that this study is not representative of results of other states and territories.
Murdoch and Denz-Penhey [74]	Australia	2007	Descriptive opinion piece	• Argues that main rural medical workforce has to be made up of contextually trained rural generalists. • Supports an academic discipline of rural and remote medicine supported through medical school, postgraduate councils and colleges • Needs to be an increased training focus on rural medical generalists from rural student recruitment to established rural career, via undergraduate education, rural pre-vocational postings, and vocational and continuing education.
Murray and Wronski [87]	Australia	2006	Descriptive opinion piece	• Describes the need to increase the medical generalist training to develop a workforce that can provide primary and secondary care.
Nixon et al. [58]	New Zealand	2007	Descriptive opinion piece	• Describes moves in NZ to establish a professional body for rural hospital generalist doctors and to recognise the role as a scope of practice.
Northern Territory Government Department of Health [27]	Australia	2016	Programme description	• Definition of RG—similar to Qld. • RG pathway offers guaranteed training places, priority rotations and structured mentoring, supervision and support. • ASTs are anaesthetics, obstetrics and emergency medicine. • ASTs under development are Internal Medicine, Surgery and Paediatrics.
Nova Public Policy Pty Ltd. [8]	Australia	2010	Government (commissioned) Report	• Examines whether the QRGP could potentially be expanded nationally. • The conclusion reached is that there are some core principles to the programme that could be adopted in all jurisdictions. • Includes a broad international literature scan.
Pashen et al. [42]	Australia	2007	Systematic review	• Comprehensive research study into a range of key components of rural generalism, including: • Definitions • Scopes of practice • Workforce supply • Education and training • Procedural skills • Funding • Safety and quality • Service provision models • Legislation • Clinical privileging
Pereria [57]	Australia	2010	Descriptive opinion piece	• Dr identifying as a Rural Generalist and the advanced skills practised (general anaesthesia, obstetrics, including forcep and caesarean sections, and in-patient and emergency care).
Queensland Health [24]	Australia	2016	Programme description	• QRGP overview. Aims to provide medical graduates with a supported training pathway to a career in rural medicine and rural and remote communities with a medical workforce. • Defines the RG and lists what the QRGP offers in terms of supported training and quarantined places. • Links to further details including background and the application process.
Queensland Health (Ernst and Young) [49]	Australia	2013	Government report	• Evaluation of the QRGP. • The study presents the strengths and criticisms of the QRGP to date and a cost analysis base on a return on investment model.
RDAA [89]	Australia	2012	Position paper	• Proposes a National Advanced Rural Training Program with a strong focus on principles underpinning a national approach to supporting rural generalist training.
Rural Health West [52]	Australia	2015	Quantitative and Qualitative study—surveys and interviews	• The capacity to practise procedural skills was the 4th highest influence on going rural. However, concerns about workload was also a negative factor about going rural. • Doctors recognised recent efforts to develop a rural generalist pathway but there was a general lack of awareness as to when and how the WA rural generalist practice pathway will be implemented.
Senate Community Affairs Committee Secretariat [85]	Australia	2012	Government report	• Examines factors affecting the supply and distribution of health services and medical professionals in rural areas. The Senate Committee stated their strong support for the Queensland RGP and recommends expansion of RG pathways.
Sen Gupta et al. [111]	Australia	2013	Descriptive opinion piece	• Responds to the Kitchener article on the QRGP • Acknowledges unintended consequences for the ‘equilibrium’ in the private sector but argues that 34 of 111 trainees in year 3 or beyond are concurrently or wholly in private practice, the same proportion as the 30% reported in 2011.
Sen Gupta et al. [48]	Australia	2013	Programme Description	• Definition provided of rural generalist as an extended medical generalist. • Background provided to Queensland programme. • Queensland pathway characterised by both training and employment reform.
Sondergeld and Nicholas [112]	Australia	1998	Quantitative and qualitative study—survey and interviews	• Indicates a clear relationship between rural GPs ceasing procedural work and the level of their indemnity premium (a barrier of the time of writing).
Stratigos and Nichols [4]	Australia	2002	Position paper	• Reforms priorities for the rural generalist workforce in: 1. Training 2. Indemnity 3. Local facilities and equipment 4. Social and financial issues 5. Retention 6. Continuing Medical Education and Upskilling • There is no evidence that outreach programmes provide a viable alternative to local procedural practice and therefore can only act as a supplementary workforce. Continuity of care is a key reason for this.
Tolhurst et al. [70]	Australia	2006	Qualitative study—interviews	• Explores factors influencing urban background medical students’ interest in rural practice. • One of the ‘work preference’ results indicated the ability to undertake procedural work in hospitals as well as provide primary GP care as an influencing factor.
Wainer [113]	Australia	2004	Quantitative study—survey	• Women make up less than a quarter of the rural general practice workforce and an even smaller percentage of the specialist rural medical workforce. Their experiences are not well articulated in research and policy on rural medical practice. • The incoming cohort of rural general practitioners has a majority of women. • Recommendations include linking female medical students with female rural doctors, matching trainees with female mentors, adequate skill development in areas important to rural practice, and ensuring a career path in rural practice.
Worley et al. [41]	Australia	2000	Qualitative study—interviews	• Overview of the Parallel Rural Community Curriculum (PRCC). • PRCC affirmed the potential role of true generalist physicians in undergraduate medical education. • The students developed a high level of competence in procedural skills and an increased confidence with patients.
North America				
Angle et al. [79]	Canada	2009	Qualitative study—secondary analysis of qualitative data	• Explored barriers experienced by physicians in providing obstetrical anaesthesia care in Ontario community hospitals that experience low volume deliveries per year. • Difficulties were greatest for FP/GP anaesthetists in rural communities due to lack of locums, the need for relevant CME, and worsening physician shortages threatening the provision of services in some rural hospitals. • Need for increased numbers of FP/GP anaesthetists and the development of formal funded networks for knowledge transfer between academic and community hospitals as means of providing supports.
Aubrey-Bassler et al. [68]	Canada	2007	Quantitative data analysis	• Authors concluded that these GPs performed caesarean sections with an acceptable degree of safety compared with specialists.
Avery et al. [114]	Canada	2014	Descriptive opinion piece	• The authors express their concern about the Privileging Standards Project and the methodology involved in the project to establish a minimum number of procedures to maintain currency. • The unintended consequence of this project could be the departure of rural generalists with skills from British Columbia with advanced skills in surgery, anaesthesia, emergency, and maternity care.
Baker et al. [115]	USA	2010	Quantitative study—survey	• Results identified a broad range of advanced skills practised by rural Family Practitioners in Idaho including obstetrics, Colonoscopy, Emergency room coverage and mental health services.
Bronstein [78]	USA	1992	Quantitative study—survey and data analysis	• This analysis distinguishes between counties with larger populations and counties with smaller populations. • Rural generalist physicians were more likely than rural specialists to have at least two of the components required to maintain obstetrics services. However more of these physicians than any other group left obstetrics over this period. • Specialists entered markets where generalists used to practise, driving the generalists to more rural areas or out of obstetrics practice altogether.
Crutcher et al. [44]	Canada	2005	Quantitative study—survey	• This study examines where Canadian family medicine graduates learned to do the procedures they perform. The findings reinforce the important role that medical schools and residency training programmes play in teaching procedural skills to family medicine residents. • They also show that rural family physicians perform a greater volume of procedures than those in urban practices.
De Klerk [18]	Canada	2013	Descriptive opinion piece	• Generalism has given way to medical specialisation in Canada (and many other countries) over the past 60 years and this does not serve the dispersed Canadian population well. • The Society of Rural Physicians is advocating for a countrywide rural curriculum in medical schools to produce a well-trained doctor with the necessary knowledge and skills to pursue a career in rural medicine.
De Klerk [31]	Canada	2014	Descriptive opinion piece	• Describes the recommendations of the Cairns Consensus Statement from the First World Summit on Rural Generalist Medicine. • Rural generalist medicine embraces the Triple C principles of The College of Family Physicians of Canada: competency-based curriculum of comprehensive care, focused on continuity of education and patient care, and centred in family medicine.
Evans et al. [116]	USA	2015	Systematic review	• Review of studies of rural colonoscopy to determine speciality types providing rural colonoscopy and the quality of these procedures. • Concludes that rural generalist physicians can safely and effectively perform colonoscopies.
Geyman et al. [60]	USA	2000	Literature review	• This review was performed to discover what has been learned from various initiatives taken by pre-doctoral and graduate medical education programmes to encourage choice and preparation for rural medical practice. • Family practice the predominant speciality upon which small rural health systems need to be based. • Rural physicians need to have procedural skills in emergency medicine, surgery, obstetrics and perhaps anaesthesia; be skilled in community medicine, have computer and business skills; and also be attuned to team and group practice.
Gordon Chaytors et al. [91]	Canada	2001	Quantitative study—survey	• More family practice graduates in rural areas performing almost all types of obstetrical care • Relatively more female than male family physicians, regardless of practice location, provide obstetrical care, including obstetrical procedures. • Recommends that curriculum for the training of Family Practitioners that intend to go rural should include more procedural and obstetric care.
Gutkin [56]	Canada	2012	Descriptive opinion piece	• Canadian Family Physician College (CFPC) decision in 2008 to approve Family Physician with Special Interest or Focused Practice (SIFP) to accredit enhanced skills that meet the Triple C curriculum standards. • The need for SIFP was particularly relevant to rural family physicians with a strong need to meet needs of their communities.
Hart [17]	USA	2000	Quantitative analysis and literature review (conference paper)	• Focus on the supply, distribution and training of generalists into rural areas, with mention of the importance of advanced skills training in developing rural generalists equipped to undertake the range of care required. • Also looks at current programmes aimed at producing a rural medical workforce and concludes that the current attempts to ensure an adequate supply of providers in underserved areas is proving problematic.
Hutten-Czapski [69]	Canada	1998	Descriptive opinion piece	• Describes some of political activity around rural obstetrics in Canada as provided by family physicians, despite increasing evidence of safe outcomes.
Hutten-Czapski [117]	Canada	2015	Descriptive opinion piece	• Argues that using statistics to support any argument that low volume obstetric care in rural areas provided by generalists equates to low quality is not supported by research. • Low-volume obstetrics has been found to be at least as safe as obstetrics practiced in big centres.
Iglesias and Hutten-Czapski [118]	Canada	1999	Descriptive opinion piece, including a literature review	• Advocates for an integrated advanced maternity care training programme for rural family physicians as a way of continuing to provide rural services in Canada in a time of a rapid decline in the availability of rural maternity services.
Imrie et al. [6]	Canada	2011	Literature review	• In recent years some family physicians have integrated additional competencies into the development of focused practices in family medicine, including areas such as emergency medicine, palliative care, elder care and rural care. • It is also stated that generalism is not just a rural and remote practice but that it is important also in urban settings.
Inglis [53]	Canada	1995	Descriptive opinion piece	• Describes the work being undertaken by a collaboration between Colleges to develop an agreed set of guidelines for the provision of surgical services delivered by GPs in rural areas. The guidelines were intended to enable the development of a training curriculum for rural surgery for GPs.
Jong [90]	Canada	2007	Descriptive opinion piece	• Small communities in Canada cannot sustain narrowly focused specialists. Instead more generalists and more rural doctors with broad and enhanced skills are required. Commitment was made in 2007 by the Canadian Medical Association to address the scarcity of generalist FP/GPs and generalist specialists and to improve access to enhanced skill sets training.
Kornelsen et al. [66]	Canada	2013	Qualitative study—interviews	• One of the solutions to doctor shortages in rural Canada is to promote the use of general practitioner surgeons (GPS). This is under threat however due to the due to the lack of interprofessional support garnered in education and practice. • Interprofessional conflict with professional boundary issues between surgeons and GPSs has prevented the increased update of the GPS role in rural Canada. • For populations of 5000–15 000, surgical services are provided locally by one or more GPS. For populations of 15 000–25 000, there is usually a specialist surgeon supported by one or more GPS (‘mixed’ model).
Lew et al. [80]	USA	2009	Quantitative study—survey	• In rural areas EDs are often staffed by primary care physicians, rather than emergency medicine trained specialists. • More than one third of the respondent physicians currently covering the ED reported that they derive greater than 40% of their income from working in it. • Respondents covering ED expressed low confidence in dealing with paediatric emergencies and highlighted a need for more training in this area.
Lockyer and Norton [54]	Canada	2005	Descriptive opinion piece	• The authors document the process involved in creating a collaborative, intersectoral approach to developing the Standards for Accreditation of Residency Training Programs arising from the need to resolve the FPA immediate needs but also has application to the support and development of FP Surgeons and FP Obstetricians across rural Canada.
MacLellan [30]	Canada	2006	Descriptive opinion piece	• Provides a view of the rural generalist as constantly moving along the spectrum between specialisation and integration. • Argues that rural Canada needs the generalist with defined competencies, constantly fluctuating between the primary, secondary and tertiary levels of care.
Maudlin and Newkirk [46]	USA	2010	Descriptive opinion piece	• Overview of Family Medicine Spokane (FMS, established as a collaborative effort by the University of Washington School of Medicine (UWSOM), four Eastern Washington community hospitals in Spokane, and the Spokane County Medical Society. • Of the 235 graduates of the FMS, 49% practice rurally (defined as a community of less than 25 000 population located more than 25 miles from a town larger than 25 000). • To increase the number of graduates going rural, the FMS Rural Training Track (FMSRTT) in Colville, was approved as an ‘experimental pathway’ of FMS. • Of the 35 graduates of the FMSRTT, 77% practice in rural communities.
Meyer et al. [119]	USA	2000	Quantitative data analysis	• Generalists were more likely to have performed a simple diagnostic procedure, perform the procedure for diagnostic and screening purposes and perform them in rural areas. • Generalists often perform less complex gastrointestinal endoscopies.
Miller et al. [93]	Canada	2012	Position paper and a literature review	• Provides an overview of current information on issues in maternity care relevant to rural populations. • Importance of collaborative practice models in rural and remote maternity care, including GP surgeons (with obstetrics) and GP anaesthetists, with support from enhanced roles for nurses and nurse practitioners. • Recommendations include expanding advanced skills training, including in caesarean section and obstetrical anaesthesia services for family physicians.
Oberai et al. [94]	Canada	2014	Descriptive opinion piece	• In rural Canada, family physicians are the main providers of maternity care. • However, fewer Canadian generalists are skilled in advanced maternity care. There has been a high rate of attrition among physicians who provide maternity care in rural areas. • If rural maternity care is to continue in Canada, rural practitioners will need training in advanced maternity care.
Ramsey et al. [45]	USA	2001	Descriptive opinion piece	• Evolution of the University of Washington School of Medicine (UWSOM) to increase generalist physicians in the region with an emphasis on rural practice. • Known as the WAMI programme after the first 4 participating states (Washington, Alaska, Montana and Idaho) and also now includes Wyoming. The WAMI programme is a rural training pipeline from undergraduate to residency with an emphasis on community practice training, including the Family Medicine Spokane (FMS) residency programme.
Rivet et al. [71]	Canada	2007	Quantitative study—secondary analysis of a population survey	• The range of procedures done by family physicians was significantly linked to job satisfaction. The larger the range of procedures, the more satisfied the physician. Rural physicians were also more satisfied than urban.
Sisler et al. [120]	Canada	2013	Quantitative study—survey	• Overview of GP Oncologists. • Whist this is not a strictly rural vocation, the role of FPs with focused practices is particularly critical in rural Canada.
Soles [59]	Canada	2015	Descriptive opinion piece	• Highlights the move away from generalism and its impact on rural communities in Canada. • Supports the principles of rural generalism in the Cairns Consensus Statement in addressing rural community need.
Thompson and Iglesias [55]	Canada	1998	Descriptive opinion piece	• Describes a proposed model of shared skill sets for the teaching and evaluation of rural generalist physicians with advanced skills. • Also raises the possibility of the establishment of a college for rural medicine in Canada as a way of preparing rural generalists with the advanced skills needed in rural areas. • Describes the need for FPs to identify skills set needed for RG advanced scopes of practice. Areas where some work has been done includes O&G and Anaesthetics. More needs to be done on areas of general \surgery and endoscopy.
Urbina et al. [72]	USA	1994	Descriptive opinion piece	• The authors discuss both the problem and various existing innovative strategies to prepare a generalist medical workforce (in family practice, internal medicine and paediatrics) with a strong focus on rural. • This article also describes the subspeciality domination of hospital based graduate medical education and the impact of that on generalist training.
Wetmore et al. [121]	Canada	2005	Quantitative study—survey	• The objective of this study was to create a list of core and enhanced procedures suitable for family medicine training. • Sixty-five core procedures and 15 enhanced procedures were identified.
Williams [32]	USA	1998	Quantitative data analysis	• Rural generalist family physician practices require different skills, are faster paced and demand more time and must deal with higher burdens of illness compared with urban practices. The higher level of hospital intensive care and obstetrical privileges and greater use of procedures by rural family physicians support these observations. • The rural family physician workforce however has continued to decline, whilst the urban rates increased.
Wootton [92]	Canada	2007	Descriptive opinion piece	• Rural physicians should be able to provide the required secondary care and also primary care in rural areas. At present, primary care is treated in education and training programmes as the main focus and this makes the advanced skills training an ad hoc add on.
Africa				
De Villiers [96]	South Africa	2004	Descriptive opinion piece	• Circumstances in South Africa call for a well-trained generalist that also includes practical/procedural clinical skills regarded by some as the domain of other specialties. The FP should be positioned as the key professional in the District Health System.
Downing [97]	Kenya	2008	Descriptive opinion piece	• Family medicine cannot just be primary care providers and the priority for physicians in Kenya is on being good generalists—which requires not only inpatient care but practising emergency surgery.
Ellis [63]	Tristan da Cunha	2008	Descriptive opinion piece	• Examples of practice and skills requirements provides insight into the scopes of practice required of a rural generalist in remote areas (Tristan da Cunha).
Hill [64]	South Africa	1995	Descriptive opinion piece	• Describes the procedural activity in the town of Kokstad—a town of 25 000 people. • Argues that primary care is not enough to attract new doctors and in state based hospitals, they need to have additional procedural skills, which makes the role more attractive. • Hill proposes more structured secondary care training programmes for generalists in procedural skills as in Australia and Canada.
Howe et al. [95]	South Africa	2013	Descriptive opinion piece	• The authors argue that expert family physician generalists are required to support primary health care as well as to provide care at the district hospital. • District hospitals, especially in rural areas, require family physicians with an extended range of skills in hospital care. • The challenge therefore for family medicine training programmes is to maximise the number of future family physicians and to re-orientate and ‘up skill’ the existing doctors for their new roles in a reengineered primary care.
Levack and Levack [5]	Tristan da Cunha	2013	Descriptive opinion piece	• Focus on the health workforce needs of a remote island, Tristan da Cunha.
Monjok et al. [13]	Nigeria	2010	Descriptive opinion piece	• Proposes a short obstetric-training programme for generalist medical officers to increase the number of skilled birth attendants in both rural and peripheral health facilities in Nigeria.
Philpott et al. [12]	Ethiopia	2014	Descriptive opinion piece	• Ethiopia’s first training programme in family medicine was launched on February 4, 2013, at the Addis Ababa University, College of Health Sciences, School of Medicine. GPs have been an important part of the health system for decades but until now there has been no postgraduate training programme for generalist physicians. The family medicine programme aims to provide such training, so that its graduates will be highly skilled comprehensive-care doctors for urban and rural areas of Ethiopia who choose generalism as a lifelong career choice. • Family physicians working in rural areas may act as consultants to other health care workers, may have greater community and public health roles, and will be able to provide emergency surgical and obstetrical services.
Reid et al. [62]	South Africa	1999	Quantitative data analysis and qualitative study—interviews (focus groups)	• Defines the role and scope of the rural generalist in South Africa as extremely wide and is often called upon to perform clinical activities ranging from primary care to emergency surgical procedures, as well as leadership roles. • Training in South Africa needs to capture the specific skills of the generalist and when the rural generalist needs to refer on for specialist care. • Indicates the need for well-planned support strategies for doctors in rural hospitals at a distance from specialist support.
Reid et al. [61]	Africa	2011	Qualitative study—interviews	• In Sub-Saharan Africa family physicians and generalist medical officers are likely to need more surgical, anaesthetic and procedural skills to provide services at the district hospital, as well as skills in consulting, mentoring and teaching to support the front line primary care workers. • Curricula should ensure that clinical training is sufficiently comprehensive to ensure competency across a broad range of diagnoses and procedures.
Thigiti et al. [47]	Kenya	2011	Programme description (conference paper)	• Overview of the Kenyan Moi University Master of Family Medicine Training Program, which aimed to address the lack of generalists in Kenya and prepares family physicians for their role as Superintendents in peripheral hospitals or as District Medical Officers. • This programme is increasing access to health care, especially for rural and poor underserved communities and is expanding into Uganda and Rwanda.
Europe				
Boerma et al. [22]	Europe	1998	Quantitative study—survey	• Procedural tasks were greater for rural GPs and for those practising at greater distance from the nearest hospital. However this was only true of western European countries and where the GP was self-employed. • The authors also identify studies that further show that in the United Kingdom and Netherlands, rural GPs undertake more procedures than their urban counterparts.
Iversen et al. [81]	United Kingdom	2002	Qualitative study—interviews	• Pressures of rural GPs having to deal with anything and everything (such as minor surgery, accident and emergency work and dispensing), due to small practice teams and considerable distance from general hospital services
Tucker et al. [82]	Scotland	2005	Quantitative and qualitative study—survey and interviews	• Medical workforce issues and falling birth rates are driving centralisation of acute obstetric and neonatal services in the United Kingdom, further limiting geographical access for remote and rural populations. Some general practitioners in this study noted that, because they no longer obtained much obstetric experience, any intrapartum care in community settings was increasingly undertaken by midwives.
Wiegers [83]	Europe	2003	Descriptive opinion piece	• In Europe the role of GPs or Family physicians in obstetrics has been in steady decline and do not get involved in high-risk obstetrical care at all.
International				
WHO [99]	International paper	2010	Position paper	• There is evidence to show that enhanced scopes of practice leads to increased job satisfaction. This resulted in recommendation B1: *Introduce and regulate enhanced scopes of practice in rural and remote areas to increase the potential for job satisfaction, thereby assisting recruitment and retention.* • Advanced procedural skills training can enhance the confidence of family medicine residents in rural areas and improve their competence.

The key findings in each theme are summarised below.

### Definition

The majority of data relevant to the definition of RGM comes from Australia, reflecting a growth of coordinated RGM pathways since 2005. Early developments include the Roma Agreement [23], which underpinned the establishment of the ‘Queensland Rural Generalist Pathway’ (QRGP), an initiative of the state health department [24]. Similar definitions are now found in the literature used by the Australian College for Rural and Remote Medicine (ACRRM) [25]; and in Australian Commonwealth, state and territory government documents [1, 26–28]. More recently, ACRRM and the Royal Australian College of General Practitioners have supported a definition of a rural generalist that reflects the Cairns Consensus Statement: a medical practitioner trained to meet the health care needs of rural and remote communities by ‘providing both comprehensive general practice and emergency care, and required components of other medical specialist care in hospital and community settings as part of a rural health team’ [29].

The application of specialised skills by the RGM is a focus of definitions in North America [30–32]. However, there are also some variations in the literature from this region. In the United States of America (USA), ‘generalism’ is often used to jointly describe family physicians, general internists and general paediatricians [33–35]. In the USA and Australia, there has been some criticism of RGM as defined in this review [36, 37], which focuses mainly on the expansion of family medicine fellowship training into specialised skills [37] or efforts to distinguish and then define generalism by rurality [38, 39].

### Pathways and programmes

This theme includes literature relevant to (i) medical school training designed to support and develop RGM and (ii) postgraduate (vocational) pathways and programmes.

#### Undergraduate medical training

Programmes supporting the development of RGM vary between countries, ranging from mature, government-funded models to new, and emerging programmes. In Australia, a medical student is not obliged to choose their speciality until they enter postgraduate (vocational) training, though there are medical school programmes supporting early-entry rural medical and generalist pathways [40–42]. A key example of this is university-based rural clinical schools [21], which emphasise rural recruitment, training in rural areas and rural graduate practice. These programmes have been shown to provide a strong foundation for attracting medical students to rural practice [43].

A Canadian study highlights the role of medical schools and residency training programmes in teaching procedural skills to rural family medicine residents [44]. The University of Washington School of Medicine (USA) established the ‘WAMI’ programme to increase generalist graduates in the region with an emphasis on rural practice [45]. This rural training ‘pipeline’ emphasises community practice training, including the Family Medicine Spokane residency programme, with specialised skills rotations [45, 46].

The Moi University Master of Family Medicine Training Program (Kenya) aims to address a shortage of generalists and prepares family physicians for roles as superintendents in regional hospitals, or as district medical officers [47]. This provides access to comprehensive health care services, especially for rural and underserved communities.

#### Postgraduate pathways and programmes

Six Australian state and territory governments have funded structured and supported prevocational and vocational RGM training pathways [1]. The QRGP offers postgraduate medical trainees:

- Advice and support services
- Access to a range of vocational and quarantined training opportunities
- Procedural and non-procedural training workshops [24]

The QRGP is supported by an industrial agreement that has enabled salaried senior medical officers with RGM credentials to access a higher salary range equivalent to staff specialists [48]. The QRGP is both a training and employment pathway that is founded on four ‘pillars’: recognition of RGM, practice value, a pathway to vocational practice and responsiveness to workforce redesign [49]. Evaluation of the programme found numerous community, workforce and economic benefits, with a cost analysis showing a 120% return on investment [49]. The evaluation also documented two criticisms of the programme: the restricted capacity for training providers to find rural placements for trainees not on the pathway and the negative impact of the programme on private general practice [49, 50]. Funded vocational RGM pathways now exist in other Australian states [26–28, 51, 52].

This degree of government administration, coordination and management of RGM programmes is unique to the Australian context. However, there are training programmes and agreements organised toward similar goals in other countries. Ethiopia’s first training programme in family medicine was established in 2013 [12] with the aim of providing postgraduate training to develop comprehensive-care generalist doctors for underserviced urban and rural areas [12]. A collaboration between the Royal College of Physicians and Surgeons of Canada and the College of Family Physicians of Canada (CFPC) developed guidelines for surgical services delivered by family practitioners (FPs) in rural areas [53]. Similarly, a shortage of rural FP anaesthetists led to the development of accreditation standards which also applied to training of FP surgeons and FP obstetricians across rural Canada [54]. Additionally, there was a call to establish a college for rural medicine in Canada to specifically prepare rural generalists with specialised skills [55]. In 2008, the CFPC approved family physicians with special interests and accredited enhanced skills that met the Triple C curriculum standards (‘continuing care centred’ in family medicine) [56]. This was particularly relevant to rural FPs, where these skills were more commonly required [56].

### Scope of practice and service models

Ideally, scope of practice is tailored to meet community needs and is responsive to a range of factors, including population size, demographics, burden of disease, access to specialist services, geography and socioeconomic status [42]. As the provision of primary health care is common to RGM internationally, the literature on scope of practice is largely focused on the additional, specialised skills provided.

The QRGP supports advanced skills training (AST) in adult internal medicine, Indigenous health, emergency medicine, paediatrics, mental health, obstetrics and gynaecology, anaesthetics and surgery [24]. The procedural skills listed here are common to the scope of practice in other states and territories across Australia and in New Zealand, in particular obstetrics and gynaecology, anaesthetics, emergency medicine and surgery [27, 28, 57, 58].

These procedural skills are also common to RGM in Canada and the USA [59, 60]. In Western European countries, the rural generalist undertakes some procedural tasks, especially in minor surgery [22]. In sub-Saharan Africa, obstetrics, anaesthetics and surgery are common skills for rural family physicians [61, 62]. In South Africa, the generalist in remote areas can also provide orthopaedic care and ENT practice [63, 64].

Whilst core procedural skills are a feature of RGM, there is also evidence of training in non-procedural tasks. The QRGP includes Indigenous health, paediatrics and mental health in the supported ASTs [24]. The Tasmanian Rural Medical Generalist Program has also identified needs in psychiatry, radiology and palliative care [51]. Palliative and elder care is also featured in Canadian RGM training [6, 65].

Discussion on scope of practice extends to models of care, including interaction between generalists and medical specialists, and the quality and safety of comprehensive care. In Canada, there is general agreement between specialist colleges that a generalist approach to procedural services in rural areas is the only feasible solution to rural medical workforce issues [66]. However, there is ongoing interprofessional debate between rural general practice and surgery about role delineation, despite it being uncommon for smaller communities to have surgical services provided by a resident specialist surgeon [66]. Kornelsen et al. (2013) claim that in communities with populations of 5000 to 15 000, surgical services are usually provided by one or more rural GP surgeons, whilst for populations of 15 000 to 25 000 surgical services are usually provided by a specialist surgeon supported by one or more GP surgeons [66]. Australian models of care are similar in that specialised skills practised by the rural generalist increase with complexity with less specialist support as rurality or remoteness increases [67]. In South Africa, there are two opposing views on the model of remote emergency care: (i) stabilisation and transportation of patients to a larger hospital and (ii) support local hospital services where the generalist can treat most cases [63].

The model of care where the generalist provides increasing specialist care proportional to remoteness is also supported by quality and safety outcomes [42]. In Canada, a study comparing caesarean section services provided by rural GPs with those of specialists concludes that rural GPs perform this procedure with an acceptable degree of safety [68]. Rural hospitals in Nova Scotia with less than 100 deliveries a year performed by rural generalists have also shown the lowest perinatal morbidity and mortality rates in the province [69]. Thompson and Iglesias (1998) conclude that there is no evidence to support exclusive skills sets given numerous quality and safety studies demonstrate identical standards for both rural generalists and urban specialists [55].

### Enablers and barriers to recruitment and retention

The ability to be trained in, and then practice, specialised skills is considered essential in successful RGM recruitment and retention. The ability to combine procedural work with primary health care is key to much rural recruitment in the Australian context [52, 70]. This, combined with the commencing salary and financial incentives offered under the QRGP, have had a positive impact on rural medical workforce recruitment [49]. In the USA, training programmes producing rural generalist graduates also emphasise comprehensive advanced skills training as key to their success [17, 60]. A study in Canada also showed the larger the range of procedures practised by a family physician, the more satisfied they were in their profession [71].

However, the trend of medical graduates toward highly specialised career choices and corresponding control of hospital-based training posts by specialists are considered threats to RGM in North America [32, 72] and Australia [73]. This also adversely affects the distribution of the overall medical workforce due to the urban-centric focus of speciality practice [73]. There are further systemic barriers for rural generalist practice in Australia, including a lack of appropriate training opportunities and support [74], complexities in maintaining and practising advanced skills, the limited availability of the supporting workforce, working hours and lifestyle factors, perceived medico-legal problems [75, 76], a lack of recognition for the rural generalist role and GPs’ reluctance to resume procedural practice once they had ceased [52, 77].

In the USA, one article identified high liability insurance premiums as a threat to viable smaller rural generalist practices, as well as limited technical facilities and the lack of an appropriate support workforce [78]. In Canada, difficult access to locums, the need for more education and training [79], low confidence in responding to paediatric emergencies and worsening physician shortages [80] are seen as the major barriers to developing the rural generalist workforce. In Europe, the pressures of providing the dual-role of primary care practitioner and specialist in rural communities [81], as well as an increasing centralisation of specialist services to larger centres, are negatively affecting RGM [82]. As a result, fewer rural GPs are practising obstetrics in Europe and it is increasingly rare for those remaining to undertake high-risk obstetric care as routine practice [83].

### Reform recommendations

The reform theme can be separated into recommendations from the literature that (i) are focused on training and (ii) have a broader workforce focus.

In Australia, recommendations for training reform include: improving linkages between Commonwealth and State training programmes; increasing support for universities committed to rural generalism; identifying new advanced skills [84]; accelerated vocational pathways into RGM; training and supporting a rural female proceduralist workforce; extending the QRGP training model into other Australian states [85]; and establishing training support networks [42, 86, 87]. The concept of a national RGM training pipeline in Australia is common to many of the training recommendations [1, 74, 88, 89]. Similarly, a USA article supported the concept of a national rural training pipeline that recruits from rural communities, provides rural placements throughout medical school, supports residencies in the rural setting and provides support in rural practice after training [17].

In Canada, the Canadian Medical Association committed to expand the number of rural generalists in training [90] and the Society of Rural Physicians have proposed developing a national rural medicine curriculum to promote the RGM workforce [31]. Other Canadian-based proposals include the establishment of a college for rural medicine [55], an extra training year with focus on procedural and obstetric care skills for family practitioners intending to work rurally [91], expanding and improving enhanced skills training programmes aligned to community need [59, 90, 92] (including advanced maternity care [93], anaesthetics, general surgery [94] and endoscopy [55, 79]).

In Africa, recommendations include increasing the number of rural generalists in training and providing a more structured secondary-care curricula across a broad range of diagnoses and procedural skills [64], similar to Australia and Canada [61, 95]. In South Africa it is recommended that more generalists with specialised skills be trained to position them as the leading health professional in the District Health System [96].

There are also recommendations for new RGM training models in many countries. Recommendations in Kenya include expanding the scope of practice for rural generalists to include emergency surgery [97]. In Japan the authors of one article advocate for the establishment of a rural generalist practitioner training programme with specialised skills, including internal medicine, gastroenterology and general surgery [98].

The literature also contains recommendations for broader workforce policy reform. A New Zealand article outlines efforts made to recognise the RGM role as a specific discipline to advance RGM practice [58]. In Australia, such recommendations include new national funding models that support the RGM pathway [42], workforce strategies aimed at recruitment and retention of rural generalists [48], supporting flexible models of practice ownership [73] and developing a national approach to recognising the rural generalist role [89]. Many of these relate to the broader agenda to develop a national RGM pathway throughout Australia [1, 8, 85] and to establish a specific role in the Commonwealth Government dedicated to this task [25].

The Cairns Consensus Statement contains policy-based recommendations under the domains of ‘Recognition, Training and Research’ for global action in RGM [2]. This is complemented by an earlier WHO recommendation to establish and regulate enhanced scopes of practice (including for Family Medicine) in rural and remote areas [99].

## Discussion

The effort to develop an internationally agreed definition of RGM and priorities for action through the Cairns Consensus Statement provides an opportunity to review global approaches relevant to RGM [2]. This is further underpinned by international health care planning, including the WHO Workforce 2030 Strategy, which aims to correct workforce supply, maldistribution and the imbalance of specialists to generalists [16].

This review found a significant body of literature relevant to the subject of RGM. However, the majority of this originates from Australia, New Zealand and North America (82 out of 102 articles). This reflects the relative maturity of, and funding allocated to, coordinated RGM programmes and pathways in these regions. The smaller volume of literature from lower-middle income countries and/or lesser developed programmes reflects a need for increasing research, support and evidence to evaluate and progress their RGM training pathways and programme design.

What literature was available from these lower income countries or those with less developed programmes does show the extent of emerging interest in RGM. Thigiti et al. (2011) describe the potential to expand the Kenyan ‘Master of Family Medicine’ training programme into Uganda and Rwanda [47] and the developing family physician role in Ethiopia, which will likely provide emergency surgical and obstetric services for those practising rurally [12]. The need for a role with procedural skills, especially in emergency medicine, obstetrics and fracture management, has also been identified in rural Nepal [100, 101], whilst in India, a trial to train rural Medical Officers in Life Saving Anaesthetic Skills was recommended for extension in response to a shortage of rural anaesthetists [102]. There are also some known early RGM programmes, including in Papua New Guinea and the Cook Islands, and some discussions occurring around RGM models in Fiji, Tonga and Zimbabwe. At the World Summit on Rural Generalist Medicine in 2017, Japan also launched its Rural Generalist programme.

This review also identified recommendations to coordinate national RGM pathways within Australia, Canada and the USA, which illustrates the need for ongoing improvements in countries where there are established programmes. Reflections on the literature identifying such improvements could also present valuable learnings for emerging programmes as they continue to build RGM models matched to local needs. Future research on commonalities and contextual differences between RGM programmes internationally (in both high and low income settings) could further understanding of best practice in RGM policy, training and delivery.

Descriptive opinion pieces were the most common form of article identified in this review (40 in total), highlighting the lack of high quality research evidence on RGM. This supports the need for more research to improve the quality of RGM-relevant data as programmes continue to develop internationally in response to ongoing rural health and health workforce needs.

## Conclusion

Developing RGM training programmes and models of practice can be a key strategy in improving health care and outcomes in rural communities around the world. This review has synthesised literature relevant to RGM, its development and implementation internationally. Whilst the majority of articles originate from Australia, Canada and the USA, there is also literature emerging from countries such as Japan, Kenya, Uganda, Rwanda, Ethiopia and India. Efforts to coordinate and strengthen RGM pathways as a response to both workforce shortages and health needs in rural and remote areas internationally are now being shared through forums such as the biennial World Summit on Rural Generalist Medicine. Scale-up of high-quality research and publication of evidence related to RGM is now required to support best practice outcomes as this momentum continues to build.

## Abbreviations

ACRRM: Australian College of Rural and Remote Medicine
FP: Family practitioner
GP: General practitioner
QRGP: Queensland Rural Generalist Pathway
RGM: Rural generalist medicine
WHO: World Health Organization
